# Supplementary material for: Does seniority always correlate with simulated intubation performance? Comparing endotracheal intubation performance across medical students, residents, and physicians using a high-fidelity simulator
Source: PLoS One. 2021 Sep 24;16(9):e0257969. doi: 10.1371/journal.pone.0257969 (PMC8462689; doi:10.1371/journal.pone.0257969)
Supplement: S1 Table — Mean and 95% CI are presented. (DOCX) [file pone.0257969.s001.docx]

**S1 Table: Time of intubation attempts and peak force applied on incisor and tongue across seniority using different devices in various scenarios.**

|  |  |  | **Seniority** | | | |
| --- | --- | --- | --- | --- | --- | --- |
|  | **Devices/ Scenarios** | | **UGY (n = 7)** | **PGY (n = 18)** | **R (n = 18)** | **VS (n = 7)** |
| **Intubation time (s)** | **Direct laryngoscopy** | |  |  |  |  |
|  |  | **Normal airway** | 25.8 [11.6 ± 40] | 38.5 [29.7 ± 47.4] | 37.6 [28.8 ± 46.5] | 33.7 [19.5 ± 47.8] |
|  |  | **Lock jaw** | 26.3 [11.9 ± 40.6] | 29.2 [20.2 ± 38.1] | 36.4 [27.5 ± 45.4] | 32.8 [18.4 ± 47.1] |
|  |  | **Rigid neck** | 91.3 [58 ± 124.6] | 61.3 [40.6 ± 82.1] | 69.3 [48.5 ± 90.1] | 34.6 [1.3 ± 67.9] |
|  |  | **Micrognathia** | 143.1 [106 ± 180.1] | 69.2 [46.1 ± 92.3] | 64.1 [41 ± 87.2] | 23.7 [-13.3 ± 60.8] |
|  | **Trachway®** | |  |  |  |  |
|  |  | **Normal airway** | 26.2 [11.5 ± 40.8] | 28.9 [19.7 ± 38] | 27.7 [18.5 ± 36.8] | 18.6 [4 ± 33.2] |
|  |  | **Lock jaw** | 24.6 [16.1 ± 33] | 24.4 [19.2 ± 29.7] | 25.7 [20.4 ± 30.9] | 18.7 [10.2 ± 27.1] |
|  |  | **Rigid neck** | 50.7 [12.7 ± 88.6] | 52.5 [28.8 ± 76.1] | 67.5 [43.8 ± 91.1] | 41.1 [3.2 ± 79] |
|  |  | **Micrognathia** | 70.7 [24.8 ± 116.6] | 79.3 [50.7 ± 107.9] | 45.9 [17.3 ± 74.5] | 75.5 [29.7 ± 121.4] |
|  | **Glidescope®** | |  |  |  |  |
|  |  | **Normal airway** | 33.7 [27.8 ± 39.6] | 26.3 [22.6 ± 30] | 30.2 [26.6 ± 33.9] | 22.5 [16.6 ± 28.4] |
|  |  | **Lock jaw** | 27.8 [18.8 ± 36.7] | 25.5 [19.9 ± 31.1] | 34.9 [29.4 ± 40.5] | 21 [12 ± 29.9] |
|  |  | **Rigid neck** | 66.7 [49.9 ± 83.5] | 42.3 [31.8 ± 52.8] | 50.3 [39.8 ± 60.8] | 29.7 [12.9 ± 46.6] |
|  |  | **Micrognathia** | 74.2 [49.8 ± 98.5] | 57.6 [42.4 ± 72.9] | 46.1 [30.9 ± 61.3] | 31.8 [7.4 ± 56.2] |
| **Force applied on incisor (N)** | **Direct laryngoscopy** | |  |  |  |  |
|  |  | **Normal airway** | 44.1 [15.3 ± 73] | 32.3 [14.3 ± 50.3] | 50.6 [32.6 ± 68.6] | 73.1 [44.3 ± 102] |
|  |  | **Lock jaw** | 35.3 [-3.8 ± 74.3] | 49.2 [24.8 ± 73.5] | 82.1 [57.8 ± 106.5] | 92.3 [53.2 ± 131.3] |
|  |  | **Rigid neck** | 53.6 [14.6 ± 92.5] | 63.1 [38.8 ± 87.4] | 121.6 [97.3 ± 145.9] | 124.4 [85.5 ± 163.4] |
|  |  | **Micrognathia** | 153.9 [106.8 ± 200.9] | 154.7 [125.3 ± 184] | 152.2 [122.8 ± 181.5] | 97.6 [50.5 ± 144.6] |
|  | **Trachway®** | |  |  |  |  |
|  |  | **Normal airway** | 18.9 [11.3 ± 26.4] | 18.2 [13.5 ± 22.9] | 16 [11.3 ± 20.7] | 15.3 [7.7 ± 22.8] |
|  |  | **Lock jaw** | 17.6 [8.2 ± 26.9] | 16.3 [10.4 ± 22.1] | 12.1 [6.3 ± 17.9] | 10 [0.6 ± 19.4] |
|  |  | **Rigid neck** | 34.4 [19.7 ± 49.2] | 26.5 [17.3 ± 35.7] | 33.8 [24.6 ± 43] | 30 [15.3 ± 44.7] |
|  |  | **Micrognathia** | 42.1 [26.2 ± 58.1] | 38.2 [28.2 ± 48.1] | 30.7 [20.8 ± 40.7] | 63.3 [47.3 ± 79.2] |
|  | **Glidescope®** | |  |  |  |  |
|  |  | **Normal airway** | 30.6 [19.5 ± 41.6] | 20.4 [13.5 ± 27.3] | 29.1 [22.2 ± 35.9] | 40.3 [29.2 ± 51.3] |
|  |  | **Lock jaw** | 32 [17.7 ± 46.3] | 21.8 [12.9 ± 30.7] | 28.8 [19.9 ± 37.7] | 39.9 [25.6 ± 54.1] |
|  |  | **Rigid neck** | 70 [39.9 ± 100.1] | 56.3 [37.6 ± 75.1] | 68.8 [50 ± 87.5] | 62 [31.9 ± 92.1] |
|  |  | **Micrognathia** | 50.9 [28.9 ± 72.8] | 58.7 [45 ± 72.4] | 70.5 [56.8 ± 84.2] | 67.9 [45.9 ± 89.8] |
| **Force applied on tongue (N)** | **Direct laryngoscopy** | |  |  |  |  |
|  |  | **Normal airway** | 59.4 [34.7 ± 84] | 68.7 [53.4 ± 84.1] | 73.7 [58.4 ± 89.1] | 92 [67.3 ± 116.6] |
|  |  | **Lock jaw** | 48.2 [21.9 ± 74.5] | 58.7 [42.3 ± 75.1] | 61.6 [45.3 ± 78] | 62.6 [36.4 ± 88.9] |
|  |  | **Rigid neck** | 41.5 [19.9 ± 63.1] | 58.8 [45.4 ± 72.3] | 63.7 [50.2 ± 77.2] | 71.3 [49.7 ± 92.9] |
|  |  | **Micrognathia** | 37.6 [12.5 ± 62.7] | 57.5 [41.8 ± 73.1] | 60 [44.4 ± 75.7] | 50.6 [25.5 ± 75.7] |
|  | **Trachway®** | |  |  |  |  |
|  |  | **Normal airway** | 22.9 [-0.7 ± 46.6] | 35.7 [20.9 ± 50.4] | 37.8 [23.1 ± 52.6] | 54 [30.4 ± 77.7] |
|  |  | **Lock jaw** | 18.9 [-5.8 ± 43.7] | 36.6 [21.1 ± 52] | 34.8 [19.4 ± 50.2] | 28.4 [3.6 ± 53.2] |
|  |  | **Rigid neck** | 24.6 [-0.1 ± 49.2] | 36.6 [21.2 ± 52] | 55.3 [39.9 ± 70.6] | 41.5 [16.8 ± 66.1] |
|  |  | **Micrognathia** | 26.3 [0.7 ± 51.8] | 44.6 [28.6 ± 60.5] | 55 [39.1 ± 71] | 50.7 [25.2 ± 76.3] |
|  | **Glidescope®** | |  |  |  |  |
|  |  | **Normal airway** | 39 [13.6 ± 64.4] | 50.1 [34.3 ± 66] | 70 [54.1 ± 85.8] | 47.2 [21.8 ± 72.6] |
|  |  | **Lock jaw** | 39.3 [13.5 ± 65.2] | 52.4 [36.3 ± 68.5] | 56.2 [40 ± 72.3] | 38.5 [12.7 ± 64.3] |
|  |  | **Rigid neck** | 49.7 [24.4 ± 75] | 63.7 [47.9 ± 79.5] | 58.7 [42.9 ± 74.5] | 62.5 [37.2 ± 87.8] |
|  |  | **Micrognathia** | 63.2 [37 ± 89.4] | 63.5 [47.2 ± 79.8] | 69.5 [53.2 ± 85.8] | 62.2 [36.1 ± 88.4] |

Mean and 95% CI are presented.
