# Supplementary material for: Does seniority always correlate with simulated intubation performance? Comparing endotracheal intubation performance across medical students, residents, and physicians using a high-fidelity simulator
Source: PLoS One. 2021 Sep 24;16(9):e0257969. doi: 10.1371/journal.pone.0257969 (PMC8462689; doi:10.1371/journal.pone.0257969)
Supplement: Attachment — Submitted filename: Response to Reviewers_edited20210703.docx [file pone.0257969.s002.docx]

Dear Professor Pasin

It is our pleasure to submit the revised manuscript “Does seniority always correlate with proficiency? Comparing endotracheal intubation performance across medical students, PGY and physicians using a high-fidelity simulator” to PLOS One for your consideration for publication.

We have fully engaged the reviewers’ comments and addressed their concerns point-by-point below. We have made the appropriate revisions and believe that the manuscript is now much improved.

Please review our changes. We look forward to hearing from you at your earliest convenience.

Kind regards

Shu-Chen Liao (on behalf of all authors)

**PLOS ONE PEER REVIEW**

**Reviewer #1:**

Introduction is too long. Most introduction should be moved in the discussion section. Please try to focus on the main problem, what is known and your aims.

In particular last sentence of the introduction should state: - Your main objective and your secondary objectives.

**Our Response:**

We thank the reviewer for this comment – and we have now tried to simplify our introduction. Up front, we state the objectives of the study as follows:

*“**The main objective of this study is to explore differences in intubation success rate and skill proficiency across different levels of seniority under various conditions, and to gain insights into invisible risks and deficiencies.”*

**Reviewer #1: methods**

Please every acronym should be explained in full at first appearance. *“We quantified intubation skills into (1) maximum applied force on incisor, (2) maximum applied force on tongue, and (3) Cormack Lehane grades when passing the endotracheal tube."* why these should quantify intubation skills? An excessive pressure on incisor is avoidable but a low force will result in undervision. How do you determine the best force to be applied? Moreover, CL grades should not be related to the operator but to the patient. I believe you should analyze number of participants that assign a wrong CL grade. why do you not use time to perform intubation?

**Our Response:**

With respect, we do take “time to perform intubation” as our primary outcome measure of intubation performance in the primary analysis. The maximum applied force on incisor and tongue, and Cormack Lehane grades when passing the endotracheal tube are additional secondary outcome measures to better understand performance. According to [Takeuchi S. et al. Longitudinal acquisition of endotracheal intubation skills in novice physicians. PLoS One. 2017;14;12(11):e0188224; GaudioRM,et al. Traumatic dental injuries during anaesthesia: part I: clinical evaluation. Dent Traumatol. 2010;26(6):459-65; Quinn JB, et al. Schieren M. et al. Comparison of forces acting on maxillary incisors during tracheal intubation with different laryngoscopy techniques: a blinded manikin study. Anaesthesia. 2019;74(12):1563-1571.], force applied on the incisor should be minimized during intubation with any techniques. Therefore, we take “applied force” as our secondary outcome to assess an individual’s intubation performance. Each participant must respond to the same scenarios with the same techniques, while the different CL grades achieved across the 12 intubations indicate their ability to. We appreciate the reviewer’s comment and will keep it in mind for the analysis of failed intubations. We have now rephrased the sentence and expanded the rationale behind our decision.

**Reviewer #1: Statistical analysis**

"Sample size was estimated using GPower 3.1 to evaluate the minimum sample size required using a two-tailed test, a medium effect size (d = 0.5), and an alpha of 0.05. " Required for what? Please state the outcome, the mean and the standard deviation (and the relative source) you used for power calculation.

success rate is not a continuous variable

**Our Response:**

We thank the reviewer for this comment. We have now refined the description of the sample size estimation that fit the same model used in the statistical analysis as follows:

*“**Sample size was estimated for a Chi-square test and a repeated measure ANOVA using GPower 3.1. As there was no prior evidence for physician seniority, estimates were set based on a large effect size of 0.5 for the Chi-square test and 0.4 for repeated measure ANOVA, with a correlation among repeated measures of 0.3, an alpha of 0.05, and a statistical power of 0.8. A sample size of 44 for the Chi-square test and 28 for ANOVA is needed to test for appreciable differences between the four independent group means (physician seniority) across 12 intubations (4 scenarios × 3 devices).”*

We agree that the individual subject’s success rate on each intubation should be a binary variable and we have modified it in our revised version.

**Reviewer #1: Results**

Two participants were excluded “because they did not complete the final assessment as instructed” what does it mean?

**Our Response:**

We have now re-phrased the sentence to be more explicit:

*“**Fifty-two learners were enrolled in the study, but two were subsequently excluded. One trainee did not accomplish the 12 intubations process, while another failed to provide informed consent.”*

**Reviewer #1: Discussion**

Other studies investigated proficiency and experience [i.e., "Assignment of ASA-physical status relates to anesthesiologists' experience: a survey-based national-study. Korean J Anesthesiol. 2019 Feb;72(1):53-59. doi: 10.4097/kja.d.18.00224. Epub 2018 Nov 14. PMID: 30424587; PMCID: PMC6369346."] Please discuss your results at the light of the other studies.

**Our Response:**

Thank you for the comments– this study reported the same finding, i.e., that experienced personnel tended to rely on their clinical experience (habit intrusions) more than the actual evidence when grading patients. We have discussed this study and some other appropriate studies in the discussion section.

**Reviewer #2: Important educational Issues:**

The authors address the topic of whether provider seniority correlates with performance of endotracheal intubation on a high-fidelity manikin that allows 4 different scenarios. From an educational perspective, the findings would be valuable if they established whether performance correlates across different manikin scenarios or across different intubation techniques? The study does not address another important topic, how performance in manikins would relate to performance in patients.

**Our Response:**

We thank the reviewer for the comments – the main focus of this study is to explore differences in intubation success rate and skill proficiency across different levels of seniority under various conditions and to gain insights into invisible risks and deficiencies, rather than to design and implement a simulation program. We have revised our introduction, discussion and conclusion sections to tie in better with our objective.

Previous study has reported that paramedics trained in endotracheal intubation based on a systematic manikin-only teaching program can attain acceptable individual success rates in actual field settings [Stratton SJ. Et al. Prospective study of manikin-only versus manikin and human subject endotracheal intubation training of paramedics. Ann Emerg Med. 1991;20 (12):1314-8.]. However, we do agree that a single study is not sufficient to document how performance on manikins would relate to performance on patients. Additionally, we have rephrased our conclusion section to open discussion of educational intervention.

**Reviewer #2: Major Issues**

1. Lines 123-135. Please provide more information on the statistical analysis. Did you perform the analysis with a repeated measures ANOVA model divided across technique and scenario? How did you account for seniority? Was the power analysis constructed for the same model used in the statistical analysis? The following two questions also indicate my confusion over the statistical analysis was performed

**Our Response:**

We have now refined the description of the statistical analysis to be more explicit and we have updated our power analysis to fit the same model used in the statistical analysis:

*“Statistical comparison was performed using a Chi-square test for the nominal dependent variable (i.e., success rate and Cormack Lehane grade) and a 4 × 4 × 3 repeated measure ANOVA with one between-subject factor (i.e., seniority) and two within-subject factors (i.e., scenario and device) for continuous dependent variables (i.e., time to intubation and force applied on incisor and tongue) with post hoc Tukey's test via IBM® SPSS® Statistics Version 26. Follow up Tukey HSD for two factor ANOVA was performed with comparison among marginal means. Degree of freedom was corrected using Greenhouse-Geisser estimates if sphericity was lacking. All effects were reported as significant at p< 0.05.”*

Physician seniority was defined as follows: medical students (final-year medical students of the formal seven-year medical school), PGY (postgraduate year one, the first year of graduate training after medical school, focusing on general medical training), residents (participants in a 4-year residency training program in emergency medicine developed by the Taiwan Society of Emergency Medicine, who have completed their one-year PGY program), and attending physicians (who have completed the residency training program in an accredited teaching hospital and are board certified in emergency medicine or surgery). These details have been added in the revised manuscript.

**Reviewer #2:**

2. Success rates are expressed as fractions with error bars in Figure 2 and Supplementary Table 1. Were means and standard deviations calculated from an individual subject’s success on each intubation expressed as a binary variable (0 or 1)? Only 1 measurement of success or failure per subject was available for each of the technique-scenario combinations in Supplementary Table 1, yielding a binary result. A continuous estimate of success rate could only be achieved for the group (sum of successes divided by group n) with no standard deviation available. Since individual success was a binary variable, a mixed effect multivariate logistic regression accounting for clustering across seniority might be a more appropriate statistical test.

**Our Response:**

We thank the reviewer for this comment – and we agree that an individual participant’s success on each intubation should be expressed as a binary variable. As the main focus of the study is the difference in intubation proficiency across different levels of seniority, we decided to use a simple method – Chi square test – to identify the difference. However, we appreciate the reviewer’s recommendation and retain it for our further investigation.

**Reviewer #2:**

3. How did you calculate means and error bars for each combination of seniority and technique in Figures 2-3. Did you calculate a success rate and mean intubation duration for each subject that combined values from the 4 scenarios? Similarly, did you average subject results across the 3 techniques for Figures 4 and 5? Was averaging across techniques or scenarios part of the statistical analysis?

**Our Response:**

Yes, the mean and error bars (i.e., ±1 SE) were calculated for each significant interaction of repeated measure ANOVA - we have added the details in the statistical analysis mentioned above.

**Reviewer #2:**

4. Supplementary Table 1 contains processed data. Other investigators could do more consequential analyses if the raw data were provided, i.e. results for each subject for all 4 scenarios, the 3 techniques, and labeled by subject seniority group.

**Our Response:**

We will gladly provide our raw data.

**Reviewer #2:**

5. The introduction should explain why the research described in the manuscript was important. What was the clinical, educational, or scientific value of evaluating performance of endotracheal intubation on mannequins as a function of operator seniority?

**Our Response:**

Thank you - we have now revised our introduction to explain the rationale of the research more clearly:

*“Since seniority is one of the requirements for performing difficult airway management, i**n cases where patients have difficult airway conditions, senior physicians often play rescuing roles. Indeed,* *plenty of guidelines suggest involving senior personnel when making decisions, diagnoses, and when performing difficult intubations. Although previous retrospective studies to evaluate the impact of wearing personal protective equipment during endotracheal intubation in the COVID-19 pandemic have reported that seniority is correlated with intubation success rate, other studies have argued that* *seniority is a poor predictor of expertise or proficiency. Expertise depends more on the volume of deliberate practice in which an individual has engaged.”*

**Reviewer #2:**

6. Furthermore, the introduction should also state the goal of the study and list the hypotheses. For example, did you expect performance to improve with seniority? Or, did you anticipate how scenario or technique might affect performance.

**Our Response:**

As mentioned above - we could not anticipate whether performance would improve with seniority. Therefore, we needed to design a study to explore their performance under various conditions (i.e., managing difficult airways using difficult devices), and to gain insights into invisible risks and deficiencies.

**Reviewer #2:**

7. Why did you compare subjects’ laryngoscopy performance on the basis of seniority, rather than experience. According to Anders Ericsson (Med Educ 2007; 41:1124), seniority is a poor predictor of expertise or proficiency. Expertise depends more on the volume of deliberate practice in which an individual has engaged. For endotracheal intubation, the number of procedures the person has performed would be a better estimate than seniority.

**Our Response:**

Thank you – and this is the reason we designed this study, as mentioned above.

*“Since seniority is one of the requirements for performing difficult airway management, in cases where patients have difficult airway conditions, senior physicians often play rescuing roles. Indeed, plenty of guidelines suggest involving senior personnel when making decisions, diagnoses, and when performing difficult intubations. Although previous retrospective studies to evaluate the impact of wearing personal protective equipment during endotracheal intubation in the COVID-19 pandemic have reported that seniority is correlated with intubation success rate, other studies have argued that seniority is a poor predictor of expertise or proficiency. Expertise depends more on the volume of deliberate practice in which an individual has engaged.”*

We appreciate the idea of “number of procedures” in determining skill proficiency and we are now designing a possible study to measure the physical and cognitive impact.

**Reviewer #2:**

8. Other studies have compared laryngoscopy performance measures among operators at different levels. Kerrey et al. Simul Healthc 2020; 15:251, Garcia et al. Br J Anaesth 2015; 115:302, and Hastings et al. Simul Healthc Dec 2 2020; PMID: 33273420 are just a few examples. The introduction or discussion could summarize appropriate studies from the literature to elucidate what is novel about the authors’ research project.

**Our Response:**

Thank you. We have summarized appropriate studies from the literature.

**Reviewer #2:**

9. Line 140. The paper currently provides no objective data to indicate whether the 4 groups (UGY, PGY, R, VS) differ in expertise with any of the intubation techniques. Do the authors have information about the number of times subjects had performed endotracheal intubation in patients, manikins, or both? In particular, information on experience with any of the Kyoto Kagaku DAM System scenarios would help establish whether the groups could be expected to differ in expertise and performance under the research conditions. The data could also be included as covariate(s) in the analysis.

**Our Response:**

# From our hospital records, the number of PGY physicians who performed tracheal intubation was about 10 cases each year. Emergency residents performed tracheal intubation in about 50-90 cases each year, with different resident seniority. Attending physicians performed 20-30 cases each year as a seniority rescue in difficult airway cases. Since medical student trainees have no physician licenses, there are very few opportunities to perform this invasive procedure except during their anesthesia training course. Regarding the experience with manikins, UGY had more practice time (about once each month) on manikins. Final year medical students have more experience on manikins because they need to pass the Medical Licensing Examination, and airway management is one of the core competencies. PGY have some regular airway training courses, and practice time on manikins is 3-5 times/ year. Residents and attending physicians have very little practice opportunity on manikins unless they are on the faculty of respiratory training courses.

**Reviewer #2:**

10. The results of a test will not differ between more proficient and less proficient individuals if the test is so difficult that everybody performs poorly. The same is true if the test is extremely easy and everybody does very well. Intubation appears quite difficult on all 4 Kyoto-Kagaku DAM System scenarios given the 44% success rate across all seniorities, the low success rate in the most senior group, and the high incidence, well over 50%, of grade 3 and grade 4 laryngeal views. An alternative explanation would be that none of the groups are proficient enough to do well on these scenarios. No data are available in the current version of the manuscript to make a conjecture one way or the other. Finally, the tests may not differ between groups because they do not actually reflect proficiency. Again, we do not have enough data to decide one way or the other. I think these points would be worth including in the discussion.

**Our Response:**

Thank you for your comments. These points are worthy of being included in the discussion.

**Reviewer #2: Minor Issues**

11. Line 60-61. The statement that accurate force measurement has been unavailable until now is misleading. Laryngoscopy forces have been measured and studied for 25 years or more (Bishop et al., AnesthAnalg 1992 74:411-4 , Hastings et al, AnesthAnalg 1996; 82:462-8) and studies are ongoing to this day.

**Our Response:**

We agree and the statement has been removed in the revision.

**Reviewer #2:**

12. Line 78-79. I don’t think one can make the statement that there is no difference in skill acquisition and retention of laryngoscopy skills between biologic models and simulation models. Skill transfer is problematic with practice on manikins and it has not been demonstrated that an individual can develop the level of skill necessary for laryngoscopy in patients solely by practicing in a simulated environment. Novices must practice on patients to become proficient.

**Our Response:**

We thank the reviewer for the comment and have now removed this controversial statement.

**Reviewer #2:**

13. Lines 98-100. The Methods should specify what manikins and scenarios were used for practice, how many attempts were made by each subject, and how it was determined that practice time was sufficient.

**Our Response:**

# Thank you, the simulators we used included“ Laerdal® Airway Management Trainer and MW13: DAM Simulator Training Model”; the training scenarios included difficult mouth openings, limitation of neck flexibility, and tongue swelling. We did not standardize the practice time before the final assessment but left it to the learners’ discretion, based on their own self-perceived readiness instead. We have now added these details into the method section.

**Reviewer #2:**

14. Line 114. The statement, “We recorded their first attempt performance for the analysis” implies that subjects attempted a technique more than once on each scenario, even though only the first attempt was analyzed. How many attempts were made? Why were multiple attempts allowed if only the first was analyzed?

**Our Response:**

We did not control the number of attempts as the study context was originally designed as a training course. Therefore, we only analyzed their first attempt performance as each learner was requested to perform at least once with a given technique under each scenario. Indeed, learners making multiple attempts may experience a practice effect and improve their performance under later scenarios. We have now added this issue to our limitations section.

**Reviewer #2:**

Line 139. Could you provide more information about how individuals in the PGY and R groups differed. Were the PGY trainees in their first year after medical school graduation, a position known as “intern” or PGY-1 in the US. Did the R group correspond to the level known as “resident” in the US?

**Our Response: (refer to Q1)**

It is not equivalent to the US system. In Taiwan, we established the 1-year PGY training program in 2011, focusing on general medicine training. PGY refers to the first year of graduate training after completing the formal seven years of medical school and getting a physician’s license. During the one-year PGY training, physicians receive four months’ internal medicine training, two months’ general surgery training, one month of emergency medicine training, one month each of pediatric- and obstetrics/gynecology training, two months in community medicine, and one month of self-selected training. After completing the PGY training course, they can apply for a resident program, which refers to a subsequent 4-6 years of residency training in different specialties.

**Reviewer #2:**

15. Tables 2-5 should have legends explaining what the columns and error bars represent and how they were calculated.

**Our Response:**

Thank you. We have now added legends to explain what the error bars represent (i.e., error bars represent ±1 SE).

**Reviewer #2:**

16. Lines 179-184 and Table 1. To my knowledge, the Kyoto-Kagaku DAM System uses an algorithm based on the manikin head and neck geometry to estimate the laryngeal view. In this study population, a high percentage of the intubations were characterized by poor views, even with the Glidescope. It would be worthwhile for the investigators to determine whether the DAM laryngeal view estimate agreed with the view determined by an expert operator over the different techniques and scenarios.

**Our Response:**

There are four scenarios in the Kyoto-Kagaku DAM System, including normal, locked jaw, rigid neck, and micrognathia. In the rigid neck scenario, the degree of neck stiffness is similar to patients with ankylosing spondylitis or patients wearing cervical collars when using direct or video laryngoscopy to perform endotracheal intubation. In the micrognathia scenario, the difficulty of tracheal management is similar to patients who received radiotherapy or who have had long-term use of betel nut, which leads to muscle fibrosis. In the rigid neck and micrognathia scenarios, instead of laryngoscopy, supraglottic airway device (SAD) or flexible fiberscope may be more suitable devices to perform intubation [Frerk, C. et al. Difficult Airway Society 2015 guidelines for management of unanticipated difficult intubation in adults. BJA: British Journal of Anaesthesia, 2015, 115(6), 827-848.]. One limitation of our study is that we should consider providing more devices like supraglottic airway device (SAD) or flexible fiberscope in future studies and provide more training following the Difficult Airway Society guidelines.

**Reviewer #2:**

17. Line 214. The Schieren reference, #38, reports that more experienced anesthetists actually generated higher peak dental forces during manikin laryngoscopy than did less experienced individuals. This is consistent with your finding. Experienced operators might accept dental force in order to accomplish a difficult intubation, realizing that the a failed intubation may risk more serious morbidity or death compared to dental trauma.

**Our Response:**

Thank you - we have now re-phrased the sentence to be more explicit and added these comments in the discussion.

**Reviewer #2:**

18. The 90-minute airway course and intubating testing involved a considerable number of procedures. Could fatigue have affected results, especially in the second half of the study with intubations requiring higher peak forces and longer durations.

**Our Response:**

We agree that fatigue could degrade learners’ performance and we have added this issue to our limitations section. Although we did not receive any complaints from learners, we could measure learners’ fatigue levels to provide a reference point for the training course design.

**Reviewer #2:**

19. Did the seniority groups differ in the amount of practice on any of the scenarios during the airway course? Could superior results by the UGY group on some scenarios reflect greater practice under those conditions in the course or prior to the study?

**Our Response:**

As mentioned above, we did not standardize the participants’ practice time – and we did not record their practice time. We have included this issue in our limitations section as practice might have an impact on the participants’ performance in the final assessment. Moreover, we agree that familiarity with manikins could be a possible explanation behind UGY’s superior results, and we have added this to our discussion section.
