# Supplementary material for: Does seniority always correlate with simulated intubation performance? Comparing endotracheal intubation performance across medical students, residents, and physicians using a high-fidelity simulator
Source: PLoS One. 2021 Sep 24;16(9):e0257969. doi: 10.1371/journal.pone.0257969 (PMC8462689; doi:10.1371/journal.pone.0257969)
Supplement: Attachment — Submitted filename: Response to Reviewers_edited_edited_20210909_Final.docx [file pone.0257969.s003.docx]

Dear Professor Pasin,

We are pleased to submit the revised version of our manuscript to PLOS One for your consideration. In order to be more explicit we have revised the title to“Does seniority always correlate with simulated intubation performance? Comparing endotracheal intubation performance across medical students, residents, and physicians using a high-fidelity simulator”.

Upon full engagement with the reviewers' comments, we have addressed their concerns point by point below. We have made all the necessary revisions and now consider it to be much better than the previous version. We have clarified the design of the study, and how we manipulated the practice sessions before the assessments. For the analysis, we have also included all first-attempt data, and we have now revised our paper to be more explicit.

Please review our changes. We look forward to hearing from you at your earliest convenience.

Kind regards

Shu-Chen Liao (on behalf of all authors)

**PLOS ONE PEER REVIEW**

**Reviewer #1:** All comments have been addressed

**Reviewer #2:**

**Reviewer #2: Major Comments**

1. The authors appear to use the term “proficiency” to mean skill at intubating mannequins. However, I generally associate intubation proficiency with competence in patients, since performing the procedure on mannequins is unimportant and uncommon for attending physicians. Therefore, the authors should define what they mean by proficiency early in the manuscript.

**Our Response:**

We thank the reviewer for this comment. The term proficient intubators refers to providers who can adjust their techniques to accommodate the conditions of patients to maintain an airway and provide ventilation quickly [1]. The most proficient intubators are associated with fast diagnoses, quality intubations, and fewer incidents [2-3]. Although previous study has assessed physicians’ intubation proficiency on a simulator [4], we agreed that it was better to illustrate the findings of the current study with the intubation performance as described in most of the existing research (e.g. [5]). We have now revised our research focus to intubation performance instead of intubation proficiency.

1.	Soar, J., et al., *European resuscitation council guidelines for resuscitation 2015: section 3. Adult advanced life support.* Resuscitation, 2015. **95**: p. 100-147.

2.	Bernhard, M., et al., *Developing the skill of endotracheal intubation: implication for emergency medicine.* Acta Anaesthesiologica Scandinavica, 2012. **56**(2): p. 164-171.

3.	Higgs, A., et al., *Guidelines for the management of tracheal intubation in critically ill adults.* British journal of anaesthesia, 2018. **120**(2): p. 323-352.

4.	Gillett, B., et al., *Skill proficiency is predicted by intubation frequency of emergency medicine attending physicians.* Western Journal of Emergency Medicine 20.4 (2019): 601.

5.	Kannaujia, A., et al., *Comparative evaluation of intubation performances using two different barrier devices used in the COVID-19 era: A manikin based pilot study.* Saudi Journal of Anaesthesia, 15(2), p.86.

2. Title. The current title would give me the wrong impression about the article because I think about intubation proficiency in terms of outcomes in patients and the paper does not investigate patient outcomes. The first sentence should be more specific, something like “Does seniority always correlate with intubation performance in mannequins?”

**Our Response:**

Thank you, we have now revised our title to be more explicit as:

*“Does seniority always correlate with simulated intubation performance? Comparing endotracheal intubation performance across medical students, residents, and physicians using a high-fidelity simulator.”*

4. Lines 35-36. I would be specific in the sentence beginning “However, when devices used . . .” I recommend, “However, VS demonstrated shorter duration than medical students using Glidescope and direct laryngoscopy.”

**Our Response:**

We have revised our result to be more specific as follows:

*“However, VS demonstrated shorter duration than medical students using Glidescope and direct laryngoscopy, whereas VS and R applied significantly more force on the incisor in the normal airway and rigid neck scenario respectively.”*

5. Lines 102-123. What was the purpose of the 90-minute practice phase? I can imagine several explanations: 1) The practice session was offered as optional training and provided an opportunity then to recruit and perform research on a large number of subjects. 2) The practice session was intended to give subjects familiarity with the equipment but was not intended as an intervention. 3) The practice session was an intervention for another study. The methods section should explain the purpose of the practice session and whether it was considered one of the experimental procedures.

**Our Response:**

The 90-minute training section phase comprised 3 sections including material review (30 minutes), technique familiarity (30 minutes), and manikin practice (30 minutes). We have presented this in Figure 1, and clarified it in the method section as follows:

*“Each session lasted about 90 minutes and comprised 3 sections: 30-minute material review, 30-minute device familiarization (i.e., practice on direct laryngoscopy, Trachway®, and Glidescope®), and 30-minute manikin practice section (Fig 1). This design offered sufficient demonstration and practice time for learners to become familiar with the devices and intubation on the manikin but was not intended as an intervention.”*

As the reviewer mentioned, the practice session was intended to give subjects familiarity with the equipment but was not intended as an intervention.

6. Lines 110-123. Why did the subjects perform 3 attempts with each modality in each scenario, if only 1 attempt in each was used for analysis. This should be explained in the Methods session.

To be frank, it looks like the experimental design and analysis plan initially involved analyzing the effects of the practice session using all 12 intubation attempts and that this plan was changed after the study was completed.

**Our Response:**

We have analyzed all 12 intubations (4 scenarios and 3 devices) as described in the section on statistical analysis. Consequently, each subject was required to attempt each modality at least once in each scenario since only the first attempt would be taken into consideration. Due to our inability to anticipate the effect of practice during assessment, we do concede that learners who attempted multiple times during assessment improvement may have been a consequence of their practice. It has been included in our limitations as described in the previous response to reviewers. In Line 295 – 298, we described:

*“Moreover, we did not control the learners’ number of attempts during the practice section and assessment; we only analyzed their first attempt at each scenario. Learners with a higher number of attempts may have benefitted from a practice effect and thereby improved their performance in later scenarios.”*

7. Line 213. The statement that “seniority correlates with success rate but not proficiency” is an incomplete and somewhat inaccurate statement of the key findings. The study does in fact show that attending physicians were more likely than medical students to intubate the mannequin trachea successfully. In addition, attending physicians were faster than medical students on Glidescope and direct laryngoscopy intubations. These are the metrics that I would most closely associate with proficiency at intubating a mannequin. The discussion should reflect this finding. The force measurements do not fit the picture the authors would expect if attending physicians’ skill exceeded that of junior subjects, i.e. less force exerted by attendings. However, I am unsure of whether force actually correlates with skill at intubating a mannequin and I am unsure whether the authors’ expectation of lower force by senior intubators is correct.

My take on the study is that it measured different metrics that the authors believe are related to skill when intubating a mannequin. Some metrics show a level of skill in the senior subjects that is lacking in the junior subjects, while other metrics (the forces) do not give the result that the authors think is consistent with greater skill in senior subjects compared to juniors.

**Our Response:**

We appreciate the concise and powerful summary of the metrics of performance in intubating manikins of varying seniority. As noted in #1, our study has been revised to focus on intubation performance instead. This discussion section begins with the main effect (i.e., the primary finding) of seniority, and then discusses the significant interaction effect later.

We have revised the discussion paragraph to include a more detailed discussion of the force issue on soft tissue as follows:

*“**As we know, the glossopharyngeal nerve is positioned superior to the anterior surface of the epiglottis, and the vagus nerve has sensory pathways running distally from the posterior epiglottis to the lower airway. The pressure of laryngoscopy during endotracheal intubation will stimulate both nerves and may cause lethal cardiovascular responses [1, 2]. Therefore, minimizing the applied laryngoscopy force could reduce the haemodynamic response, as well as the local tissue trauma associated with intubation [3]. Accordingly, the laryngoscopy force applied to the soft tissues of the oral mucosa should be reduced as the physician's seniority and competence in the quality of their care increases.”*

1.	Kovac AL. *Controlling the hemodynamic response to laryngoscopy and endotracheal intubation.* J Clin Anesth. 1996 Feb;8(1):63-79. doi: 10.1016/0952-8180(95)00147-6.

2.	Akbar AN, Muzi M, Lopatka CW, Ebert TJ. *Neurocirculatory responses to intubation with either an endotracheal tube or laryngeal mask airway in humans.* J Clin Anesth. 1996 May;8(3):194-7. doi: 10.1016/0952-8180(95)00228-6.

3.	Russell T, Khan S, Elman J, Katznelson R, Cooper RM. *Measurement of forces applied during Macintosh direct laryngoscopy compared with GlideScope® videolaryngoscopy.* Anaesthesia. 2012 Jun;67(6):626-31. doi: 10.1111/j.1365-2044.2012.07087.x.

8. Line 284. The most significant limitation in this study is the failure to control the extent of subjects’ practice in the 90-minute session before the test phase. The practice session constitutes an uncontrolled intervention, even if not intended that way. Subjects practiced until they perceived that they were ready to move on, not in the controlled fashion if practiced were allowed to some pre-set number of replicates or until a standardized performance was reached. The variability in practice could have substantially increased variability among subjects, diminishing the study’s power toward distinguishing differences across junior and senior groups of subjects. The limitations section should acknowledge this potential problem.

If the authors changed the experimental design or analysis plan after the study was complete, they should acknowledge this as a limitation as well.

**Our Response:**

The term “practice time” refers to the number of attempts during the practice section instead of time for practice. The time for practice before assessment was controlled, with each learner allowed to practice for 30 minutes as mentioned before (refer to #5). However, the number of attempts during the 30-minute practice section were not manipulated. Therefore, we didn’t record their intubation performance and frequency during the practice section. We have now rephrased the misleading term “practice time”. We agree that the number of attempts may have affected learners’ later performance, and have now addressed the problem as a limitation more explicitly.

9. The Cormack Lehane grade view measured by the MW11 system estimates the line of sight view obtained during the intubation attempt. However, operators generally obtain a grade 1 or grade 2 view with the Glidescope and Trachway camera chips, even on difficult airways. Operators have would have no reason optimize the line-of-sight view since they have the video view, and usually pay no attention to the line of sight. Thus, the MW11 measurements of line-of-sight view are irrelevant for intubation with the Glidescope and Trachway and the analysis represented by Table 1 is meaningless. The authors may want to re-think incorporating the Cormack and Lehane view as a measure in this paper.

**Our Response:**

Thank you for your comments. We have reconsidered the significance of Cormack Lehane grade performance of various devices in this study. As the reviewer stated, the line-of-sight view from a video laryngoscope (both Glidescope and Trachway) is from the camera lens of the video chipset, just like the view of an operator lying in front of the throat. Cormack Lehane grade differs from the original grading concept. Therefore, we have removed the part of the analysis involving both video laryngoscopes and re-presented the results.

10. Title. The term “PGY” will be confusing to the American medical audience because PGY residents are physicians. More specificity might be possible by changing the phrase in the title to “medical students, residents, and attending physicians.”

**Our Response:**

Thank you - we agree this is a much clearer title for the general audience and have revised it in the latest version.

11. Line 26. I recommend changing the phrase “skill proficiency” to “other skills.”

**Our Response:**

We have revised this phrase in line 26, and line 76 also. Thank you.

12. Lines 27 and 80. The authors do not define and I do not understand the term “invisible risks and deficiencies.” I would remove the term and any further discussion because it is not closely related to the goal of evaluating how parameters measured during mannequin intubation vary among individuals with different levels of seniority.

**Our Response:**

We have now deleted this term and the further discussion.

13. Line 55. Degree of success is most importantly evaluated by whether the tube is placed in the trachea. I would re-phrase the sentence to, “Intubation may also be evaluated by . . .”

**Our Response:**

Thank you for your comments, and we have now rephrased as follows:

*“The degree of success of intubation is traditionally evaluated by the time required and the achievement of both-lung ventilation. Intubation may also be evaluated by additional indicators such as applied force and glottic view that could affect patient outcome.”*

14. Lines 77-78. The main objective should be changed to “explore differences in intubation success rate and other measurements during intubation of the Kyoto Kagaku MW11: difficult airway management (DAM) simulator evaluation system.” (consistent with item 11 above)

**Our Response:**

Thank you, and we have revised it.

15. Lines 102-110. Did subjects practice on direct laryngoscopy, Trachway, and Glidescope during the 90-minute practice phase? Please specify in the text at this point.

**Our Response:**

Thank you. We have now specified these three devices:

*“Each session lasted about 90 minutes and comprised 3 sections: 30-minute material review, 30-minute device familiarization (i.e., practice on direct laryngoscopy, Trachway®, and Glidescope®), and 30-minute manikin practice section (Fig 1).”*

16. Lines 131-132. In item 16 of my last review, I was interested in whether the Kyoto-Kagaku MW.11 system measurement of Cormack and Lehane view had been validated, i.e. was the view accurate when compared to the view reported by a trained operator performing the laryngoscopy. The authors did not answer this question

**Our Response:**

The Kyoto-Kagaku MW.11 difficult airway management simulator was developed at Waseda University in 2006 to serve as an active training device and as an advanced evaluation tool for surgical instruments and medical procedures [1, 2]. The simulator was built based on the concepts of a Japanese anesthesia textbook (Tracheal Intubation Visual Manual of Clinical Basic Techniques) [3]. However, we were unable to discover any English literature describing the process of Cormack and Lehane grade verification [4]. This has been addressed as a study limitation.

1.	Y. Noh et al., Development of the airway management training system WKA-4: For improved high-fidelity reproduction of real patient conditions, and improved tongue and mandible mechanisms. 2011 IEEE International Conference on Robotics and Automation. 2011:1726-31.

2.	Y. Noh et al., Development of the airway Management Training System WKA-5: Improvement of mechanical designs for high-fidelity patient simulation. 2012 IEEE International Conference on Robotics and Biomimetics (ROBIO). 2012:1224-9.

3.	Kazuyoshi Aoyama(2009). *Tracheal Intubation Visual Manual of Clinical Basic Techniques.* Japan：Yodosha.

4.	I. Okuyama et al., *Reproduction and verification of airway difficulties using airway management training system WKA-5.* Journal of Japan Society Computer Aided Surgery.Vol13. No3. 2011.P224-225.

17. Line 128, Line 33: The proportion of both lungs ventilated is not reported later in manuscript. The term could be deleted line 128 and in the abstract.

**Our Response:**

Thank you, we have now deleted this parameter.

18. Line 218. Reference 17 is described as a report of a correlation between seniority and intubation speed, but the paper describes emergency patient intubations and the abstract of this paper does not mention speed.

**Our Response:**

Thank you, we have updated our reference:

Kim, Sin Young, et al. "How much experience do rescuers require to achieve successful tracheal intubation during cardiopulmonary resuscitation?." *Resuscitation* 133 (2018): 187-192.

19. Line 223. Contrary to the text, reference 18 reports that the literature is not consistent on whether video-laryngoscopy hastens or prolongs intubation.

**Our Response:**

Thank you, we have updated our reference:

Griesdale, Donald EG, et al. "Glidescope® video-laryngoscopy versus direct laryngoscopy for endotracheal intubation: a systematic review and meta-analysis." *Canadian Journal of Anesthesia/Journal canadien d'anesthésie* 59.1 (2012): 41-52.

20. Line 269. I don’t think you should to ascribe shortcomings to the senior physicians based on their performance. The 4 mannequin scenarios appear to be quite difficult and the attendings achieved the highest success rate of any of the groups, even though one presumes that they had no training on these scenarios. Other groups generated lower dental forces, but this may have contributed to their lower success rates. I would suggest replacing “shortcomings” with “greater dental force” or something more specific.

**Our Response:**

Thank you, we have rephrased the term to be more explicit.

21. The phrase, “across seniority,” is repeated in the title to Supplementary Table

**Our Response:**

Corrected, thank you.
